# Symmetric patterns with different luminance polarity (anti‐symmetry) generate an automatic response in extrastriate cortex

**DOI:** 10.1111/ejn.14579

**Published:** 2019-10-03

**Authors:** Alexis D. J. Makin, Giulia Rampone, Marco Bertamini

**Affiliations:** ^1^ Department of Psychological Sciences University of Liverpool Liverpool UK; ^2^ Department of General Psychology University of Padova Padova Italy

**Keywords:** electroencephalography, event‐related potentials, lateral occipital complex, sustained posterior negativity, symmetry

## Abstract

People can quickly detect bilateral reflection in an image. This is true when elements of the same luminance are matched on either side of the axis (*symmetry*) and when they have opposite luminance polarity (*anti‐symmetry*). Using electroencephalography, we measured the well‐established sustained posterior negativity (SPN) response to symmetry and anti‐symmetry. In one task, participants judged the presence or absence of regularity (Regularity Discrimination Task). In another, they judged the presence or absence of rare colored oddball trials (Colored Oddball Task). Previous work has concluded that anti‐symmetry is only detected indirectly, through serial visual search of element locations. This selective attention account predicts that the anti‐symmetry SPN should be abolished in the Colored Oddball Task because there is no need to search for anti‐symmetry. However, this prediction was not confirmed: The symmetry and anti‐symmetry SPN waves were not modulated by task. We conclude that at least some forms of anti‐symmetry can be extracted from the image automatically, in much the same way as symmetry. This is an important consideration for models of symmetry perception, which must be flexible enough to accommodate opposite luminance polarity, while also accounting for the fact anti‐symmetry is often perceptually weaker than symmetry.

## Graphical abstract

Visual symmetry (where luminance is matched) and anti‐symmetry (where luminance is reversed) both generate an ERP called the sustained posterior negativity (SPN). The SPNs are similar when participants discriminate regularity or dot color. This shows that some forms of anti‐symmetry, for instance in dot patterns, are extracted from the image automatically, without selective attention.

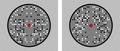

**Abbreviations**
ANOVA: analysis of variance
EEG: electroencephalography
ERP: event‐related potential
fMRI: functional magnetic resonance imaging
ICA: independent components analysis
LIMO: linear modeling toolbox for EEG
LOC: lateral occipital complex
RAGU: randomized graphical user interface
SPN: sustained posterior negativity
SSVEP: steady‐state visual‐evoked potential
TANOVA: topographic ANOVA
TMS: transcranial magnetic stimulation

## 1 INTRODUCTION

Sensitivity to visual symmetry has been observed in mammals, birds, fish, and insects (Benard, Stach, & Giurfa, 2006; Delius & Nowak, 1982; Grammer, Fink, Møller, & Thornhill, 2003). Psychophysical experiments have established that symmetry perception is efficient (Barlow & Reeves, 1979) and that reflectional symmetry is particularly salient (for reviews, see Treder, 2010; Tyler, 1995; Wagemans, 1995).

Because symmetry requires a match between elements at different spatial positions, the case when such elements differ is particularly interesting (Morales & Pashler, 1999). Stimuli with a mismatch of the symmetrically positioned elements are often referred to as anti‐symmetry. We believe that it is useful to reserve the term anti‐symmetry to cases where the mismatch is along a dimension that has poles. Here, we focus on luminance anti‐symmetry, where elements are lighter/darker relative to the background (Wenderoth, 1996). A type of mismatch related to that of luminance is color, and this has also been studied recently (Gheorghiu, Kingdom, Remkes, Li, & Rainville, 2016). Another important case is contour polarity: where the convex/concavity coding of contours can be matched or mismatched (Baylis & Driver, 1995; Bertamini & Wagemans, 2013; van der Helm & Treder, 2009).

**Figure 1:**
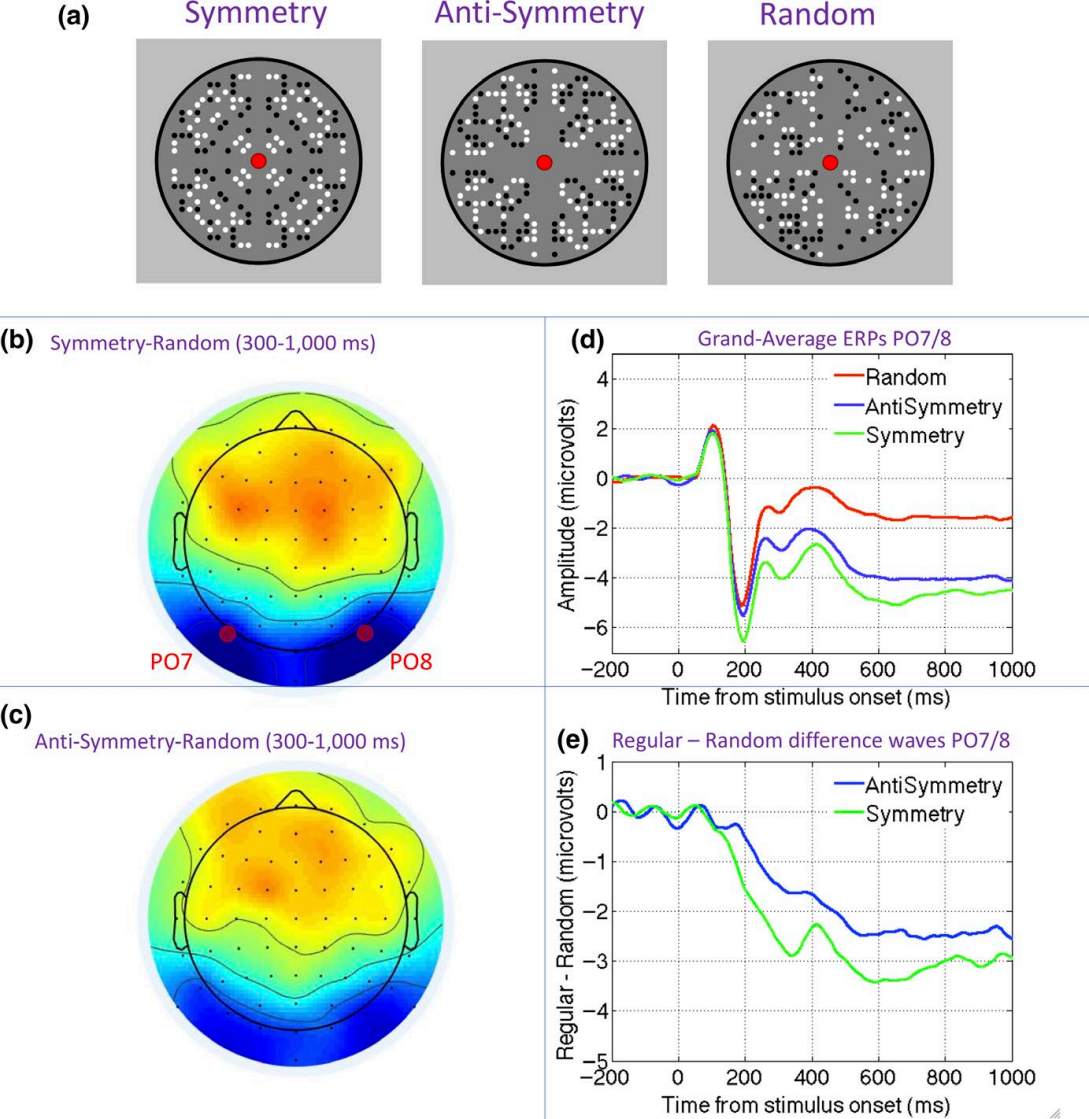
The Sustained Posterior Negativity (SPN) response to symmetry and anti‐symmetry. (a) Examples of symmetry, anti‐symmetry, and random patterns. (b) Topographic difference map generated by symmetry random comparison in the 300‐ to 1,000‐ms interval. (c) Topographic difference map generated by anti‐symmetry random in the 300‐ to 1,000‐ms interval. (d) Grand‐average waveforms from the PO7/PO8 electrode cluster in symmetry, anti‐symmetry, and random conditions. (e) Symmetry and anti‐symmetry SPNs as a difference wave. Note that symmetry and anti‐symmetry both generate a comparable SPN in terms of latency and topography, but the amplitude is slightly reduced (i.e., less negative) for anti‐symmetry. Materials in this figure are from Experiment 4 in Makin et al. (2016) [Colour figure can be viewed at http://wileyonlinelibrary.com]

In the case of luminance symmetry, symmetrically positioned elements are matched in terms of luminance (e.g., black dots paired with black, or white paired with white), while in the case of luminance anti‐symmetry, elements are opposite (black paired with white, or white paired with black, Figure 1a). Psychophysical studies have shown that anti‐symmetry discrimination is inferior under certain conditions (Wenderoth, 1996). In particular, anti‐symmetry discrimination thresholds are selectively increased when density is high, when energy at high spatial frequencies is removed, when stimuli are presented in the visual periphery, or when gray‐scale level variability is increased (Gheorghiu et al., 2016; Huang & Pashler, 2002; Mancini, Sally, & Gurnsey, 2005; Rainville & Kingdom, 1999; Saarinen & Levi, 2000; Wenderoth, 1996; Zhang & Gerbino, 1992).

In the last two decades, the nature of the human brain response to symmetry has also been investigated (Bertamini & Makin, 2014; Bertamini, Silvanto, Norcia, Makin, & Wagemans, 2018; Cattaneo, 2017). Functional MRI work has identified a symmetry‐related activation in extrastriate regions such as lateral occipital complex (LOC) and V4, both when participants engage in active symmetry discrimination or in secondary tasks unrelated to symmetry (Keefe et al., 2018; Kohler, Clarke, Yakovleva, Liu, & Norcia, 2016; Sasaki, Vanduffel, Knutsen, Tyler, & Tootell, 2005).

Event‐related potential (ERP) methods have also been used to record the extrastriate symmetry response (Jacobsen & Höfel, 2003; Makin et al., 2016, 1). After the visual evoked potential, there is a long interval where amplitude at posterior electrodes is lower for symmetrical patterns (Höfel & Jacobsen, 2007; Jacobsen & Höfel, 2003; Makin, Wilton, Pecchinenda, & Bertamini, 2012; Norcia, Candy, Pettet, Vildavski, & Tyler, 2002). This *Sustained Posterior Negativity* (SPN) component typically begins around 200 ms after stimulus onset, and amplitude scales with the salience of different kinds of regularity (Makin et al., 2016; Palumbo, Bertamini, & Makin, 2015). The SPN for symmetry is generated automatically, even when the participants make judgments about other visual dimensions, such as color (Makin, Rampone, & Bertamini, 2015), the number of closed regions (Makin, Rampone, Wright, Martinovic, & Bertamini, 2014), or the presence of infrequent features (Höfel & Jacobsen, 2007; Makin, Rampone, Pecchinenda, & Bertamini, 2013).

We know anti‐symmetry generates a similar, but slightly reduced SPN (Makin et al., 2016; Wright, Mitchell, Dering, & Gheorghiu, 2018, Figure 1b–e). Although Oka, Victor, Conte, and Yanagida (2007) documented an odd‐harmonic steady‐state visual‐evoked potential (SSVEP) response to anti‐symmetry under passive viewing conditions, no previous work has compared SPN responses to symmetry and anti‐symmetry under different task conditions on the same participants. Therefore, we do not know whether the SPN response to anti‐symmetry is automatic and task‐independent, like the SPN response to symmetry.

Some psychophysical work indirectly predicts that symmetry and anti‐symmetry SPNs should dissociate and behave differently in the face of experimental task manipulations. Mancini et al. (2005) used checkerboard patterns and concluded that symmetry is automatically extracted by image filtering, but people only discover anti‐symmetry indirectly, via a serial visual search of element positions (c.f. Treisman & Gelade, 1980). In support, Mancini et al. (2005) found that anti‐symmetry discrimination deteriorated whenever the visual search strategy was thwarted. They concluded:

The results reported here strongly suggest that under the present experimental conditions sensitivity to symmetry and anti‐symmetry does not generally arise from similar mechanisms. Thresholds for the detection of symmetry and anti‐symmetry diverge as a function of check size, spatial frequency content, greyscale range and eccentricity. Thresholds for symmetry detection are relatively unaffected by any of our manipulations and this suggests the existence of low‐level mechanisms that are prepared to detect symmetry at a range of scales. We find that anti‐symmetry is only detected when there are few items in the display and these items have binary grey levels. These conditions suggest the need to compare individual items in the display, and hence the involvement of selective attention. (Page 2159).

van der Helm and Treder (2009) used different stimuli (and used the broader term ‘anti‐regularity’ to refer to both anti‐reflection and anti‐repetition), but drew similar conclusions about the role of selective attention in anti‐symmetry detection. Their participants judged whether inward‐facing contours of two solid black shapes were reflected. Contours on the outer sides of the shapes were not relevant for the task, but could be either congruent or incongruent with the inner ones. The authors reasoned that if congruence with task‐irrelevant outer contours facilitated performance, then the relationship between these two outer contours must have been coded by the visual system. They found a facilitation effect for task‐irrelevant symmetrical contours, but not for task‐irrelevant anti‐symmetrical contours. They thus concluded that anti‐symmetry is *not* coded automatically. van der Helm and Treder (2009) thus agreed with Mancini et al. (2005) in this respect:

…detection of regularity is part of the visual system's intrinsic encoding, whereas detection of anti‐regularity is not. It suggests further that detection of anti‐regularity requires higher cognitive strategies involving selective attention. (page 2758).

Both Mancini et al. (2005) and van der Helm and Treder (2009) noted that their conclusions about the role of selective attention in anti‐symmetry detection may not generalize beyond the checkerboards or solid shapes they used in their experiments. However, their results demonstrate that there are some cases where anti‐symmetry can only be detected with selective attention, so the role of selective attention in anti‐symmetry perception remains an open question.

To summarize, these selective attention accounts claim that symmetry is extracted from the image automatically whatever the participants task, but in contrast, anti‐symmetry is discovered with higher cognitive strategies involving selective attention and serial visual search of element locations. Presumably, these strategies would only be deployed when detection of anti‐symmetry is a task requirement. We therefore examined the SPN response to symmetry and anti‐symmetry as a function of task.

**Figure 2:**
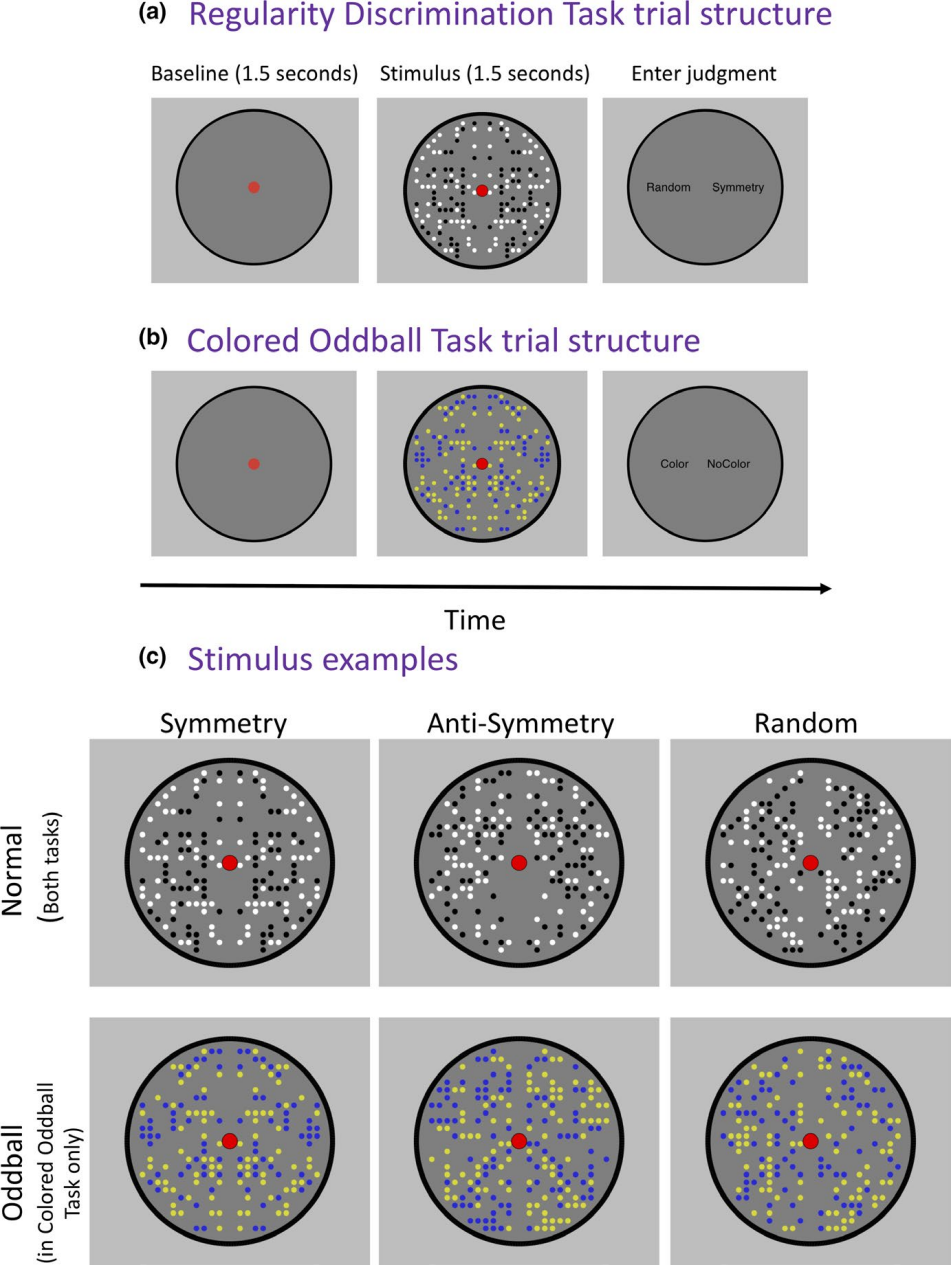
Procedure and stimuli. (a) Trial structure in the Regularity Discrimination Task. Each trial began with a 1.5 s baseline, followed by a 1.5‐s pattern presentation. The pattern could be symmetry (25% of trials), anti‐symmetry (25%), or random (50%). The patterns were always black and white. Finally, after the pattern disappeared, participants entered their binary judgment in a non‐speeded way. The task was to enter the correct judgment (‘Symmetry’ for either symmetry or anti‐symmetry, or ‘Random’ for random). (b) Trial structure in the Colored Oddball Task. Again, each trial began with a 1.5‐s baseline, then a 1.5‐s pattern presentation, and then the participants entered their binary judgment in a non‐speeded way. The patterns could be black and white (83.3% of trials) or infrequent yellow and blue colored oddballs (16.7%). The participants’ task was to enter the correct judgment (‘No Color’ for the frequent black and white patterns, or ‘Color’ for the infrequent Colored oddballs). (c) Example stimuli from both tasks. In the Regularity Discrimination Task, all stimuli were black and white (upper row). In the Colored Oddball Task, there were *additional* colored oddball trials presented (lower row). It is important to note that these patterns shown in this figure are just examples; in the experiment, each trial involved a novel pattern [Colour figure can be viewed at http://wileyonlinelibrary.com]

Specifically, we compared the SPN for symmetry and anti‐symmetry in two tasks (Figure 2). In our *Regularity Discrimination Task* (Figure 2a), participants used two buttons to classify the patterns as “Symmetry” (meaning either symmetric or anti‐symmetric) or “Random”. In the *Colored Oddball Task* (Figure 2b), the same black and white stimuli were used (Figure 2c upper), but we added a small proportion of oddball trials where the patterns were blue and yellow (Figure 2c lower). On each trial, participants classified the patterns as “Color” or “No Color”.

We used the same 1‐fold symmetry and anti‐symmetry dot patterns as Makin et al. (2016) because these stimuli were found to generate an SPN response in during explicit regularity discrimination. We predicted that the symmetrical patterns would produce a similar SPN in the Regularity Discrimination and Colored Oddball Tasks. Conversely, if the selective attention account applies to our dot stimuli, then the SPN for *anti‐symmetry* should be present in the Regularity Discrimination Task, but reduced or abolished in the Colored Oddball Task.

## 2 METHOD

### 2.1 Participants

A group of 22 participants with normal or corrected‐to‐normal vision (self‐reported) were tested (two left‐handed, two males, aged 18–30). Participants were reimbursed with course credit or travel expenses. The experiment had local ethics committee approval (PSYC‐1011009) and was conducted largely in accordance with the Declaration of Helsinki (although the study was not pre‐registered, which is required by point 35 of the 2008 revision).

### 2.2 Apparatus

The apparatus was the same as that used in our previous SPN research (Makin et al., 2012). Participants sat in an electrically shielded and darkened room, 140 cm from and 40 × 30 cm CRT monitor with a 60 Hz refresh rate. Head position was stabilized with a chin rest. Continuous Electroencephalography (EEG) data were recorded using the BioSemi Active‐2 system (Amsterdam, Netherlands) from 64 scalp electrodes arranged according to the international 10–20 system. Band pass filters during recording were set at 0.16 and 100 Hz, and sampling rate was 512 Hz.

### 2.3 Stimuli

Stimulus generation was based on the anti‐symmetry experiments in Makin et al. (2016). Patterns were generated in Python using open‐source PsychoPy software (Peirce, 2007). The code is available on open science framework on this link: https://osf.io/yjg9q/, and example stimuli are shown in Figure 2c.

**Figure 3:**
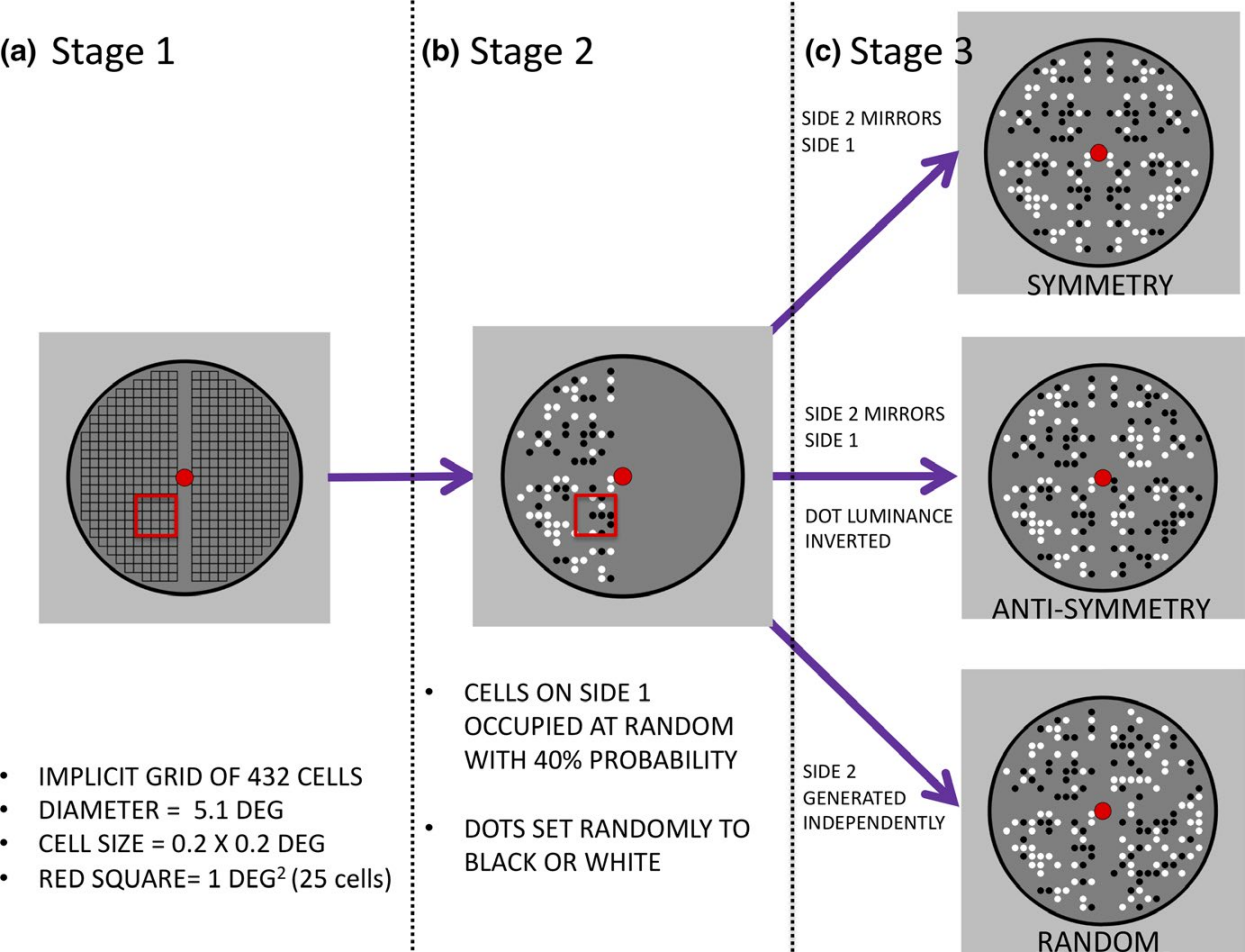
Schematic of the stimulus construction algorithm. The implicit grid (stage 1) was populated in a probabilistic way, so each cell had a 40% chance of being occupied with a black or white element (stage 2). The relationship between left and right halves then determined symmetry, anti‐symmetry, or random (stage 3). Participants only saw the final product of the construction algorithm. The red square in a and b illustrates the size of 1 square degree. The stimulus algorithm is available on Open Science Framework (https://osf.io/yjg9q/), along with a ‘Stim Maker.py’ script that generates and saves example patterns as .PNG image files [Colour figure can be viewed at http://wileyonlinelibrary.com]

The important stimulus control principle was that the *average* number of black and white dots was the same in for symmetry, anti‐symmetry, and random. In this way, we controlled some low‐level stimulus features statistically. However, the way that the stimuli were constructed resulted in trial‐by‐trial variability. Understanding the stimulus construction algorithm helps explain the nature of this variability. For illustration, consider the generation of a symmetrical pattern. At stage 1, the program created an implicit grid of 432 cells, with 216 either side of the vertical reflection axis (Figure 3a). At stage 2, each cell in the grid had a probability of being occupied with a black or white dot (Figure 3b). Finally, at stage 3, the relationship between left and right patterns was set (Figure 3c). The probability of occupation for each cell was set by a density parameter, set at 40% (to understand the density parameter, think that if density was 100%, every cell would be occupied by a dot, but if density was 0%, there would be no dots anywhere). When the density was set at 40%, the *probability* of each cell being occupied is 40%, and consequently, 40% of the available cells were occupied *on average*. The average dot number was thus 172.8. However, the number of dots on each trial varied, and *SD* for dot number was around 12.9. For random patterns, the mean occupancy was the same, but the *SD* was at around 9.3. Furthermore, each dot had an equal probability of being black or white, and the proportion could therefore vary. On average, there were an equal number of black and white dots.

The diameter of the black perimeter circle was 5.1° of visual angle. The implicit grid area where dots were permitted to fall covered 17.28 square degrees. Dots were also prevented from landing on the midline. In the grid area where dots could fall, average density was approximately 10 elements per deg^2^ (see red square in 3b).

### 2.4 Procedure

Trial structure is shown in Figure 2a (Regularity Discrimination Task) and 2b (Colored Oddball Task). Each trial began with a 1.5 s blank baseline period, followed by a 1.5 s pattern presentation. A red fixation dot was present throughout both intervals. After the stimuli disappeared, participants entered a binary judgment with the A and L keys of a standard computer keyboard (‘Symmetry’ or ‘Random’ in the Regularity Discrimination Task; ‘Color’ or ‘No Color’ in the Colored Oddball Task). The response cue locations indicated which button to use to enter a particular judgment. Response mapping switched unpredictably between trials, so participants could not anticipate which button would be used to enter their judgment until they saw the response screen.

Table 1 shows the distribution of trials in each task and condition. In the Regularity Discrimination Task, there were 72 repeats of the symmetry condition, 72 repeats of the anti‐symmetry conditions, and 144 repeats of the random condition, giving 288 trials in total. Half the trials required a ‘Symmetry’ response (meaning that they were either symmetric or anti‐symmetric), and half required a ‘Random’ response.

**Table 1:** Characteristics of trials in the Regularity Discrimination and Colored Oddball Tasks

Stimulus	Color	Correct response	Duration(s)	*N* trials
Regularity Task				
Symmetry	Black/White	Symmetry	1.5	72
Anti‐symmetry	Black/White	Symmetry	1.5	72
Random	Black/White	Random	1.5	144
Colored Oddball Task				
Symmetry	Black/White	No Color	1.5	72
Anti‐symmetry	Black/White	No Color	1.5	72
Random	Black/White	No Color	1.5	144
Symmetry	Yellow/Blue	Color	1.5	18
Anti‐symmetry	Yellow/Blue	Color	1.5	18
Random	Yellow/Blue	Color	1.5	18

In the Colored Oddball Task, the same type of black and white patterns were shown; however, there were an additional 54 colored oddballs (giving 342 trials in total). The colored oddballs had yellow and blue dots (Figure 2c). There were an equal number of symmetry, anti‐symmetry, and random colored oddball trials. At the end of each trial, participants entered a judgment of Color or No Color. The correct answer was No Color on 83.33% of the trials, and Color on just 16.67% of the trials.

The trials were divided into nine blocks. Each block included the same distribution of trials per condition as the experiment as a whole. The trials were presented in a randomized order within each block. Before each task, a single practice block was included. To control for task order effects, half the participants completed the Regularity Discrimination Task first, and half completed the Colored Oddball Task first.

### 2.5 EEG analysis

Electroencephalography analysis was conducted offline using the eeglab 13.4.4b toolbox in MATLAB 2014b (Delorme & Makeig, 2004). Raw data was first referenced to a scalp average, and low‐pass filtered at 25 Hz with the eeglab IIRFILT plug‐in. The data were then downsampled to 128 Hz and divided into −1 to +1.5 s epochs, with a −200 to 0 ms baseline.

Blink, eye movement, and other high‐amplitude artifacts arising from extra‐neural sources were removed manually with independent components analysis (ICA, Jung et al., 2000). In the Regularity Discrimination Task, an average of 9 ICA components were removed from each participant (min = 5, max = 12). After ICA, we removed trials were amplitude exceeded +/− 100 μV at any electrode (approximately 9% from each condition). This left an average of 65 trials per participant in the symmetry condition, 65 trials per participant anti‐symmetry condition, and 131 trials per participant in the random condition. In the Colored Oddball Task, an average of 8.77 components were removed (min = 2, max = 15) and around 13%–15% trials were excluded, leaving an average of 62 trials per participant in the symmetry condition, and 61 trials per participant in the anti‐symmetric and anti‐symmetry condition, and 124 trials per participant in the random condition. Trials were not excluded from ERP analysis if participants made an incorrect judgment. Oddball trials were not included in SPN analysis of the Colored Oddball Task.

The SPN was analyzed at PO7 and PO8 electrodes, from 300 to 1,000 ms post‐stimulus onset. These parameters were chosen a priori, based on previous work (Makin et al., 2016). A 2 × 3 repeated‐measures ANOVA [Task (Regularity Discrimination, Colored Oddball) × Regularity (symmetry, anti‐symmetry, random)] was used to examine effects on SPN amplitude. Greenhouse–Geisser correct factor was used to adjust degrees of freedom when the assumption of sphericity was violated. None of the 18 ERP variables deviated significantly from normality according to the Shapiro–Wilk test (*p *>* *.175).

In addition to the ERP analysis based on a priori electrode choice, we ran mass univariate analysis, which computes a multilevel pairwise comparison at each electrode and time point. This was conducted using the hierarchical linear modeling for EEG (LIMO) MATLAB toolbox (Pernet, Chauveau, Gaspar, & Rousselet, 2011; Pernet, Latinus, Nichols, & Rousselet, 2015). We also ran topographic ANOVA (TANOVA; Koenig, Kottlow, Stein, & Melie‐García, 2011) that compares topographic maps using randomization statistics. LIMO and TANOVAs confirmed that our conclusions were not problematically dependent on electrode choice.

## 3 RESULTS

### 3.1 Behavioral results

In the Regularity Discrimination Task, participants were better at identifying symmetry than anti‐symmetry (error rates 0.03 vs. 0.14). In the Colored Oddball Task, performance was near ceiling in both conditions (error rates 0.01 and 0.03 in normal and oddball trials).

### 3.2 ERP results

**Figure 4:**
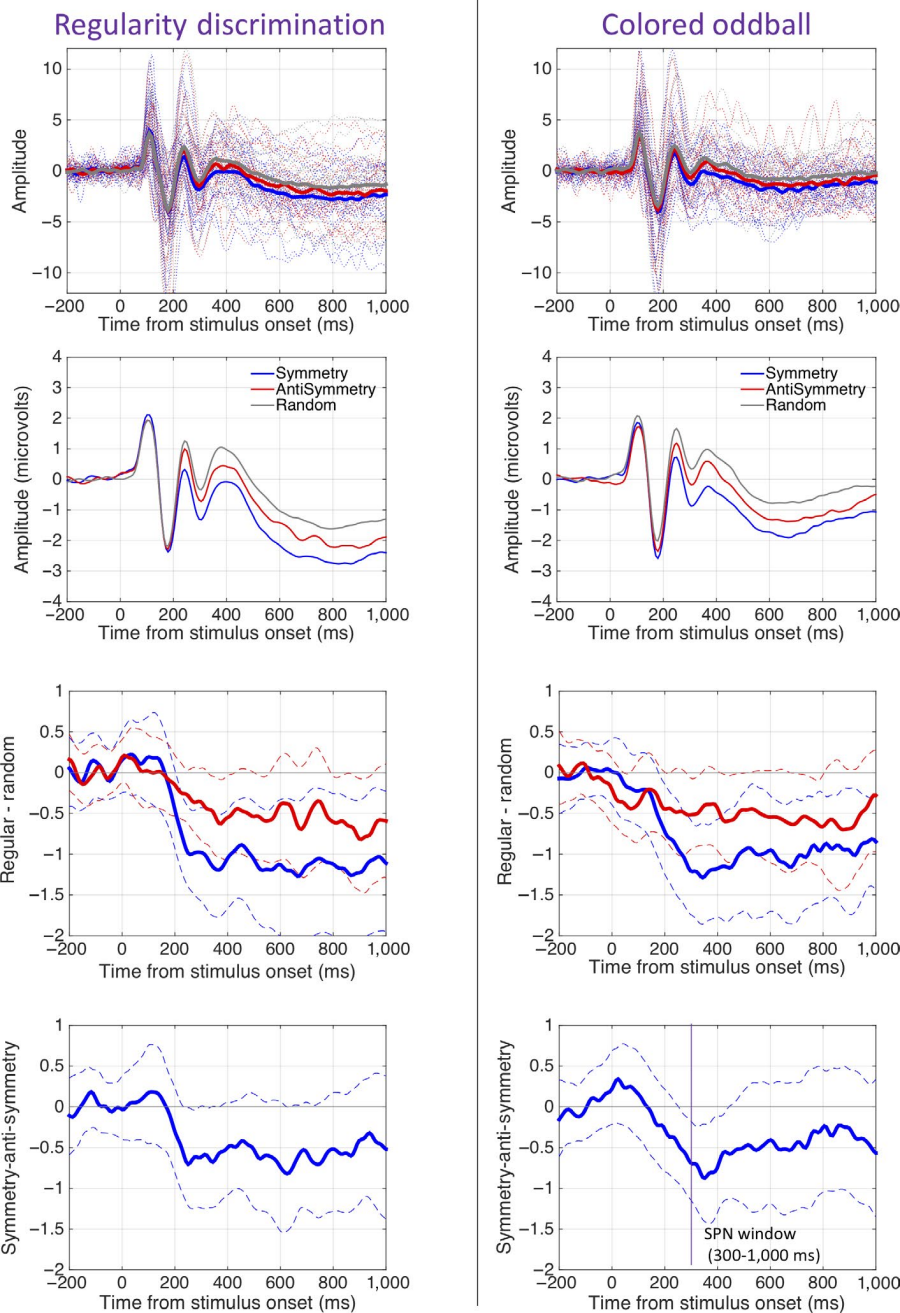
SPN waves. The left column shows results from the Regularity Discrimination Task, and the right column shows results from the Colored Oddball task. The top row shows grand‐average ERPs from electrode cluster PO7/PO8 with individual participant traces in the background. The second row shows these ERPs again on a different scale, without individual traces. The lower rows show 95% CI around the grand‐average difference waves. When CIs cross zero, the difference wave is significant at the .05 level (this was only achieved consistently for the symmetry SPN) [Colour figure can be viewed at http://wileyonlinelibrary.com]

The posterior SPN was similar in the Regularity Discrimination and Colored Oddball Tasks (Figure 4). Symmetry generated a larger SPN than anti‐symmetry in both tasks. A 2 × 3 repeated‐measures ANOVA [Task (Regularity Discrimination, Colored Oddball) × Regularity (symmetry, anti‐symmetry, random)] found a main effect of Regularity, *F*(1.450, 30.446) = 12.531, *p* < .001 partial η^2^ = .374, but no main effect of Task, *F*(1, 21) = 1.297, *p* = .268 and no Task × Regularity interaction, *F*(2, 42) = 0.036, *p* = .964.

The symmetry wave was significantly more negative than the random wave, *F*(1, 21) = 15.867, *p* = .001, partial η^2^ = .430. The symmetry wave was significantly more negative than anti‐symmetry wave, *F*(1, 21) = 6.806, *p* = .016, partial η^2^ = .245, and the anti‐symmetry wave was also more negative than the random wave, *F*(1, 21) = 12.291, *p* = .002, partial η^2^ = .368. Additional analysis with Task order as a between‐participants factor found no evidence that this influenced the results.

The topographic difference maps in 5 suggest that the SPN was stronger over the right hemisphere. We therefore ran exploratory analysis with Hemisphere (left vs. right) as an additional factor (using electrode cluster P7, P9, PO7, PO3, and right‐sided homologues). There were a main effect of Regularity, *F*(2,42) = 17.399, *p* < .001, partial η^2^ = .453 and a Hemisphere × Regularity interaction, *F*(2, 42) = 3.395, *p* = .043, partial η^2^ = .139. The interaction resulted from weaker effect of Regularity in the left hemisphere, *F*(2, 42) = 8.396, *p* = .001, partial η^2^ = .286 than in the right hemisphere, *F*(2, 42) = 18.371, <.001, partial η^2^ = .467. Crucially, there were no interactions with Task in any analyses which included Hemisphere. Therefore, the hemispheric asymmetry does not complicate the most theoretically interesting results of our experiment.1There has been some concern about the use of non‐transformed data in hemispheric comparisons (König et al., 2015). However, the data used in this analysis were normally distributed (Shapiro–Wilk tests, *p* > .175) and applying the recommended ASINH transform did not change the results.


**Figure 5:**
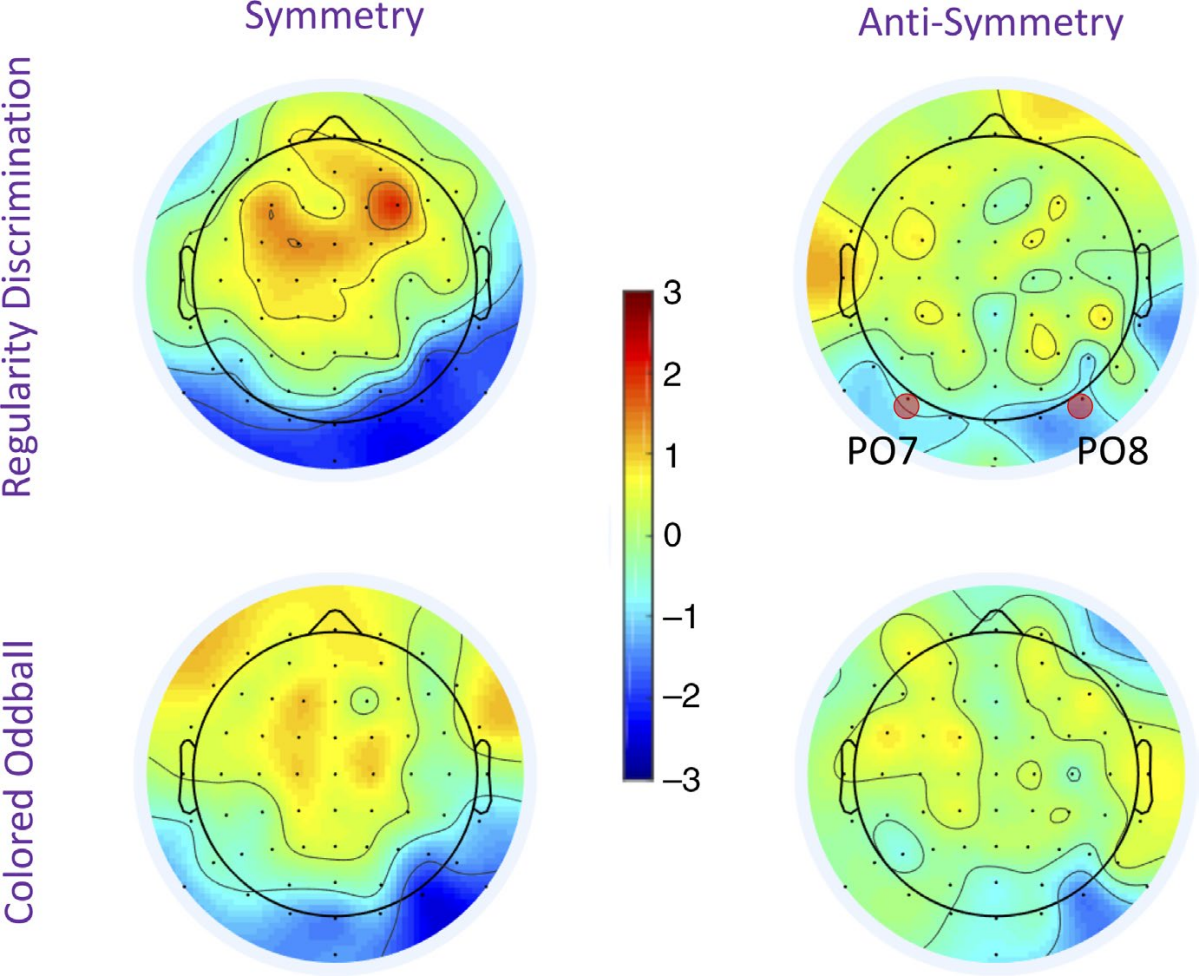
Sustained posterior negativity (SPN) topoplots. Topographies were taken from data averaged over the 300‐ to 1,000‐ms interval. The color scale represents the amplitude of the difference from the random condition. Consequently, the SPN appears as blue at posterior electrodes. The SPN was stronger for symmetry than anti‐symmetry, but comparable in both tasks. It can also be seen that the SPN was right lateralized in both tasks [Colour figure can be viewed at http://wileyonlinelibrary.com]

Unsurprisingly, the grand‐average SPN waves were not replicated in all participants individually. However, SPN frequency was identical in both tasks (confirming their general similarity in a different way). In both tasks, 18/22 participants generated a symmetry SPN (*p* = .004, binomial test) and 15/22 participants generated an anti‐symmetry SPN (*p* = .134). Only 12/22 the participants had a stronger SPN in symmetry than anti‐symmetry conditions (*p* = .832).

**Figure 6:**
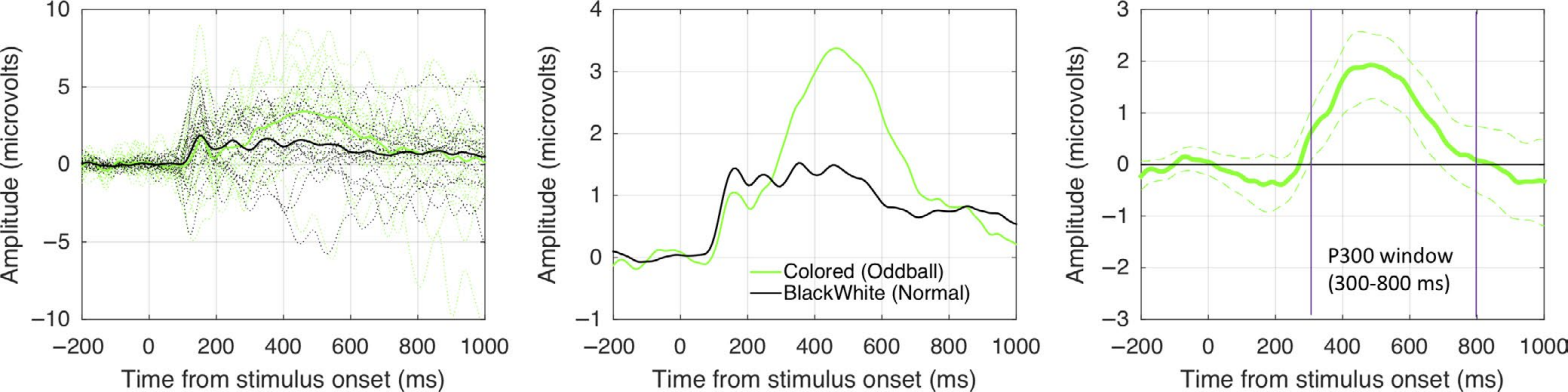
P300 generated by infrequent colored oddballs. Panels show grand‐average ERPs from electrode cluster P1, PZ, and P2, with individual participant traces in the background (left), the same ERPs on a different scale without individual traces (center) and P300 as a difference wave with 95% CI (right). The P300 is not essential for this research, but confirms that participants were engaging in the Colored Oddball Task in the expected fashion [Colour figure can be viewed at http://wileyonlinelibrary.com]

We did not include the relatively infrequent colored oddball trials in the analysis of the SPN. However, we found that oddball trials generated a P300 wave posterior central electrodes (P1, PZ, and P2) from in the 300–800 ms window, *t*(21) = 5.114, *p* < .001; Figure 6. This confirms that our Colored Oddball Task was cognitively similar to those used in previous oddball EEG studies.

### 3.3 Confirmation of the null hypothesis

Our most interesting SPN result was the *absence* of a Task × Regularity interaction. However, it is problematic to base theoretical conclusions on the absence of a significant effect. The traditional ANOVA method gives the probability of obtaining the observed data given the null hypothesis (pD|H0), NOT the probability of the null hypothesis being true given the observed data (pH0|D). We therefore used Bayesian alternatives to null hypothesis significance testing to estimate pH0|D (Masson, 2011). For the interaction term, this provided a pH0|D estimate of 0.956, and consequently, PH1|D was estimated as just 0.044. This confirms that the SPN data are a much better fit to the null hypothesis: *namely no Task × Regularity interaction*. In comparison, the null hypothesis that there is no main effect of Regularity on SPN amplitude is unlikely given the data (pH0|D) = 0.052).

### 3.4 Mass univariate analysis

How should researchers select electrodes and time points that best capture ERP effects of interest, without double‐dipping and thus inflating familywise error rate? These are important methodological issues in neuroimaging research generally (Kriegeskorte, Simmons, Bellgowan, & Baker, 2009). The analysis above was based on a priori choice of electrodes and time windows (PO7 and PO8, 300–1,000 ms). On the upside, the a priori approach avoids double‐dipping, but, on the downside, it leaves nearly all the ERP data unanalyzed. We thus ran exploratory mass univariate analysis of all electrodes and time points from −200 to 1,000 ms using the hierarchical linear modeling for EEG toolbox in MATLAB (“LIMO”, Pernet et al., 2011). This procedure applies a multilevel statistical test to each time point and electrode and thereby allows visualization of whole data set (Martinovic, Jennings, Makin, Bertamini, & Angelescu, 2018).

A 2D array of t values from uncorrected pairwise comparisons is shown in each panel of 7. All non‐significant comparisons (*p* > .05) are masked gray. In these plots, the SPN appears as blue/green at posterior electrodes. It can be seen that the SPN was then strongest in symmetry conditions (top row) where it appears as a cluster of significant comparisons, particularly in right posterior electrodes.

**Figure 7:**
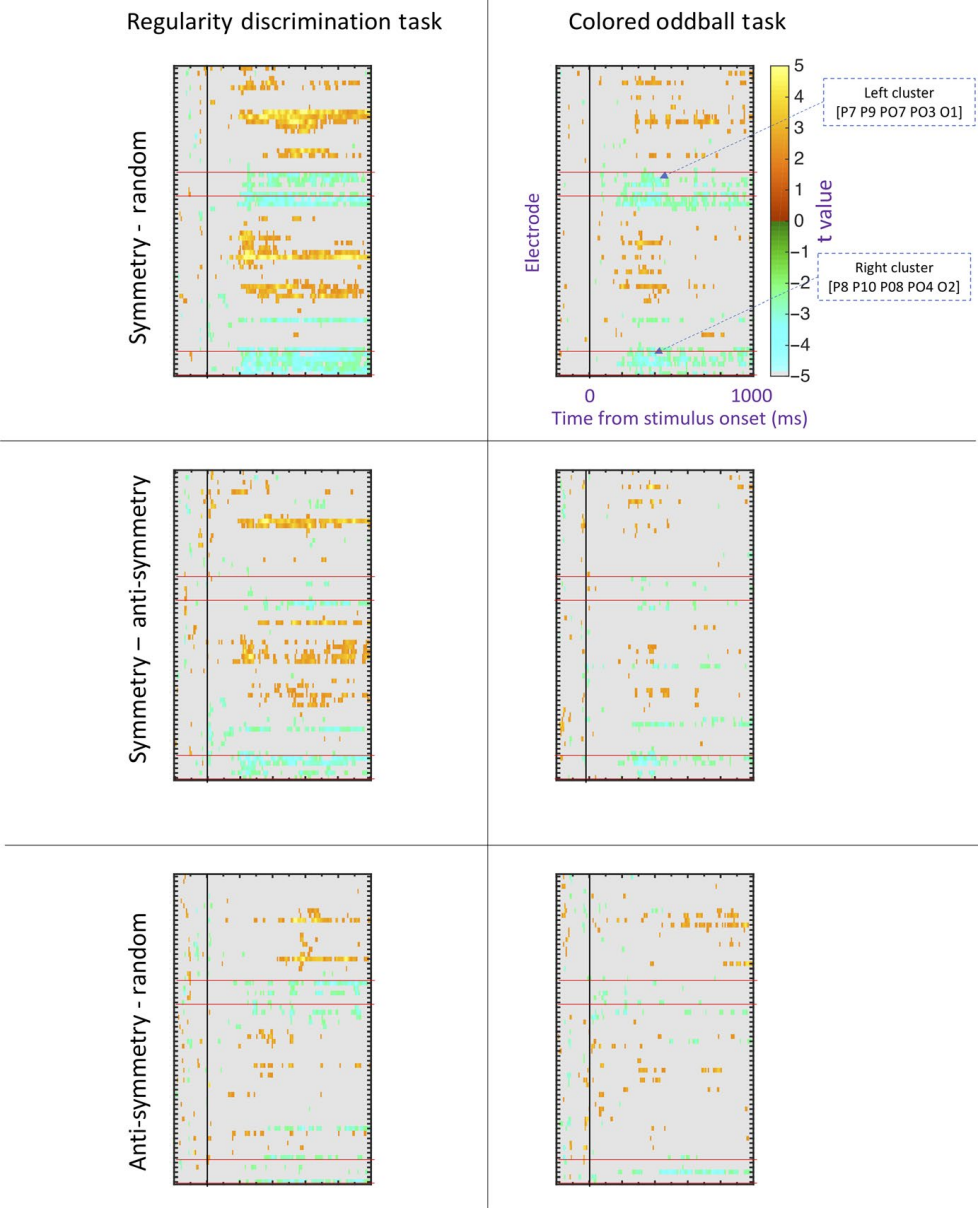
Mass univariate analysis. The left column shows results from the Regularity Discrimination Task, and the right column shows results from the Colored Oddball task. Conventions are the same in all six panels, but labels and annotations are only included in the top right. The color scale shows t values from a multilevel pairwise comparison. These are uncorrected for familywise error, so we would expect a 5% false positive rate (5% of the area to be colored). The red bands indicate posterior electrodes used in the between‐hemisphere analysis. The most robust differences were found in the right posterior electrodes when comparing symmetry to random (see top left panel) [Colour figure can be viewed at http://wileyonlinelibrary.com]

### 3.5 Topographic ANOVA

Another approach to multidimensional EEG analysis is *topographic ANOVA* (TANOVA). Recent work has also applied TANOVA to the SPN (Wright et al., 2018). This procedure assesses whether differences between topographic maps are statistically significant. TANOVA was implemented with *Randomized Graphical User interface* software (RAGU, Koenig et al., 2011), which uses randomization statistics to estimate whether observed topographic differences are greater than chance level (*p* < .05). TANOVA was based on the same pre‐processed and averaged‐over‐trial data as the standard ERPs.

**Figure 8:**
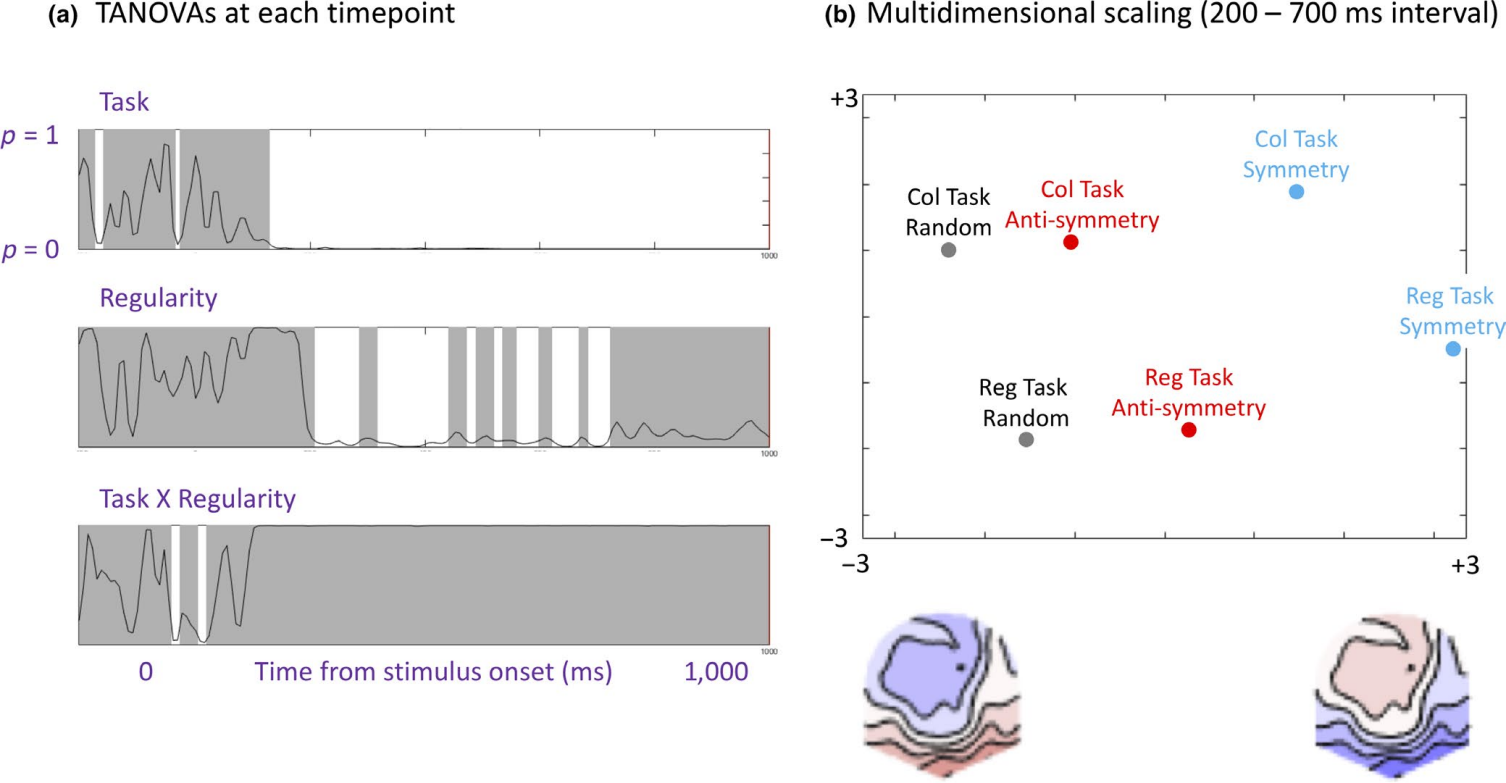
Topographic ANOVA. (a) Task × Regularity topographic ANOVA was applied to each time point from −200 to 1,000 ms from stimulus onset. Significant intervals are marked white; non‐significant intervals are marked gray. (b) Multidimensional scaling plot showing the projection of the six mean topographies onto optimized 2D results space. This maps the difference between topographies, so data points close together come from conditions with similar topographies and vice versa. Schematic topographies below the *X* axis are not real data, but represent the spatial distribution of the first principal component [Colour figure can be viewed at http://wileyonlinelibrary.com]

The crucial question here was whether the topographic differences between symmetry, anti‐symmetry, and random conditions interacted with Task. Figure 8a shows *p* values from successive TANOVAs applied to each time point from −200 to 1,000 ms. There were many time windows with a main effect of Task, beginning at approximately 150 ms. There were also frequent main effects of Regularity in the interval between 200 and 700 ms. However, there was no Task × Regularity interaction at any time point during this interval. This supports our claim that differential brain responses to symmetry, anti‐symmetry, and random patterns were not fundamentally altered by Task. To examine this further, we ran a TANOVA on data from the average interval from 200 to 700 ms. There were main effects of Task (*p* = .003) and Regularity (*p* = .004) but no interaction (*p* = 1).

The multidimensional scaling (MDS) plot in Figure 8b illustrates the topographic distribution of our ERPs in a different way. The data points in Figure 8b represent grand‐average topographies projected optimized 2D space from principal components analysis (see Koenig et al., 2011 for formal details). To understand these results intuitively, note that if two data points are relatively close, the grand‐average topographies are similar, while if two data points are far apart, the grand‐average topographies are different. Also note that data points near the left represent cases where the topographic map is more like the left schematic topoplot and vice versa. Figure 8b first reconfirms that random conditions were more positive at posterior electrodes (like the left schematic topoplot) while symmetrical conditions were more negative at posterior electrodes (like the right schematic topoplot). More importantly, Figure 8b shows that data points representing symmetry, anti‐symmetry, and random topographies were arranged in the same way in both tasks (although tasks differ on the second dimension).

## 4 DISCUSSION

We found that the SPN was larger (i.e., more negative) for symmetry than anti‐symmetry. However, all SPN waves were very similar in the Regularity Discrimination Task and the Colored Oddball Task. This finding is not consistent with the selective attention account of anti‐symmetry perception (van der Helm & Treder, 2009; Mancini et al., 2005). If anti‐symmetry SPNs are generated indirectly, via serial visual search of element locations, then the anti‐symmetry SPN should be reduced in the Colored Oddball Task, where such searching was not necessary. While the conclusions of Mancini et al. (2005) and van der Helm and Treder (2009) remain valid for their checkerboard and solid shape stimuli, our results suggest that they do not apply to the anti‐symmetry dot patterns used here.

Wright et al. (2018) reported that symmetry and anti‐symmetry dot patterns generated a comparable sequence of stable topographic microstates, suggesting that the same sequence of perceptual and cognitive processes were recruited in both cases. This aspect of Wright et al. (2018) also contradicts the selective attention account. Furthermore, Oka et al. (2007) found an odd‐harmonic SSVEP response to low‐density anti‐symmetry dot patterns during passive viewing. Finally, the fact that anti‐symmetry generates an SPN at all (rather than some very different pattern of scalp potentials related to employment of serial search by the dorsal stream) seems inconsistent with the selective attention account again (Makin et al., 2016). We thus conclude the visual system treats anti‐symmetry dot patterns like symmetry dot patterns and that anti‐symmetry is be extracted from the image automatically by the extrastriate network, whatever the participant's task.

We acknowledge that there may be other forms of anti‐symmetry which are not captured by the visual system's intrinsic coding, and are only discovered indirectly through serial visual search (van der Helm & Treder, 2009; Mancini et al., 2005). However, this does not apply to the dot patterns used in recent SPN research (Makin et al., 2016; Wright et al., 2018). Element density and presence of substructures are likely to be key determinants of automatic anti‐symmetry perception. In future work, it would be interesting to replicate our experiment with checkerboards and solid shapes. We predict that the anti‐symmetry SPN may be selectively reduced during a Colored Oddball Task with these stimuli.

The SPN was stronger over the right hemisphere in both tasks. Right lateralization of the SPN has been observed before, albeit inconsistently (Bertamini & Makin, 2014). Furthermore, recent multivoxel pattern analysis studies have found higher symmetry decoding probability from right extrastriate regions, suggesting the symmetry signal is stronger in the right hemisphere (Van Meel, Baeck, Gillebert, Wagemans, & Op de Beeck, 2019), and TMS disruption of right LOC is more costly for symmetry discrimination (Bona, Herbert, Toneatto, Silvanto, & Cattaneo, 2014). Bringing these results together, it seems that both symmetry and anti‐symmetry discrimination is mediated by extrastriate networks, which are weakly right lateralized.

### 4.1 Alternative explanations for results

It is possible that participants engaged in some spontaneous visual search for anti‐symmetry during our Colored Oddball Task. However, this could only explain our results if spontaneous searching for anti‐symmetry were deployed on nearly every trial. Furthermore, spontaneous search would presumably be more prevalent when the Colored Oddball Task was presented second, after practice on the Regularity Discrimination Task. However, there were no order effects. Consequently, we do not think the results can be plausibly explained by spontaneous search in the Colored Oddball Task.

Participants did not have to explicitly discriminate anti‐symmetry *from symmetry* in our Regularity Discrimination Task (instead they discriminated both kinds of symmetry from random—a binary classification). A three‐way classification task might have increased SPN amplitude further and lead to differences from the Colored Oddball Task. However, we do not think this is likely. Consider that the discrimination of anti‐symmetry from random was not trivial—error rate was 14%. Therefore, classifying the anti‐symmetry patterns as a type of symmetry presumably took some effort. Nevertheless, the anti‐symmetry SPN was no larger than in the Colored Oddball Task, where this effort was not required. This suggests, albeit indirectly, that anti‐symmetry SPNs would not be enhanced further if participants performed more effortful three‐way classification task.

### 4.2 Why does anti‐symmetry generate a weaker SPN?

As with previous studies by Makin et al. (2016) and Wright et al. (2018), our anti‐symmetry SPN was reduced compared to the symmetry SPN. There are at least three plausible explanations for this, and these explanations are not mutually exclusive.

First, as surmised by Wenderoth (1996), symmetry perception mechanisms do not always treat each element in isolation. Instead, the system works with perceptually grouped aggregates of elements, or substructures, whenever such grouping is possible (Csathó, van der Vloed, & van der Helm, 2003). Indeed, Locher and Wagemans (1993) concluded that global symmetry is derived from grouped pattern information, and these prior groupings serve as ‘input primitives in the construction of the global percept’ (page 582). Sometimes the properties of substructures would interact with symmetry and anti‐symmetry differentially (this has been shown in other cases, for instance, while repetition can be enhanced by substructures, reflection can actually be weakened, Csathó et al., 2003). We predict that if substructures were made more salient, for example by element tessellation or high density, then the difference between symmetry and anti‐symmetry SPNs would increase.

A second consideration is that the visual system groups elements according to luminance as well as symmetry (Wagemans et al., 2012). These grouping principles might interact. For symmetrical patterns, these two grouping principles are congruent (e.g., we have a black symmetry and a white symmetry). However, for anti‐symmetrical patterns, the grouping principles are incongruent. Grouping by luminance might first need to be inhibited in order to detect the positional symmetry in anti‐symmetrical patterns. Failure to inhibit grouping by luminance could disrupt discovery of symmetrical correspondences and thus reduce the anti‐symmetry SPN (although see Wright et al., 2018).

A third alternative involves the distinction between first‐ and second‐order visual channels. Element position information may be carried by first‐order, luminance sensitive channels, which respond differently to black and white elements on a gray background. Element position information can also be carried by second‐order, contrast sensitive channels, which respond in the same way to black or white elements on a gray background (Brooks & van der Zwan, 2002; Tyler & Hardage, 1995). For symmetry, both first‐order and second‐order channels carry useful element position information. For anti‐symmetry, only second‐order channels are available to carry useful position information. The same basic idea can be described in terms of visual filters: first‐order visual filters extract aligned blobs from symmetrical patterns only, while second‐order filters extract blobs from symmetrical and anti‐symmetrical patterns (see figure 1 in Mancini et al., 2005 for an illustration of this). When evaluating these models, one should be aware that binary distinction between first‐ and second‐order channels or filters is a potentially problematic oversimplification (Carandini et al., 2005). Nevertheless, the perceptual weakness of anti‐symmetry could be explained (at least simplistically) by selective abolition of useful first‐order element position information.

These three explanations for the perceptual weakness of anti‐symmetry overlap and may sometimes reflect superficial differences in wording or emphasis. Furthermore, as explicitly stated by most anti‐symmetry researchers, the contribution of such factors probably depends on element density and may work differently for dot patterns, checkerboards, or solid shapes (Mancini et al., 2005).

### 4.3 Relationship with previous work

Some recent work has found that SPN amplitude is independent of element luminance or color. Martinovic et al. (2018) found that the SPN was similar for color‐defined, isoluminant stimuli, and luminance‐defined, achromatic stimuli. However, in this study, elements in symmetrically corresponding positions always had the *same* color and luminance properties. This is different from anti‐symmetry, where elements in symmetrical locations have opposite color or luminance properties.

In another recent study, Wright et al. (2018) compared conditions with 50% symmetrical and 50% randomly arranged noise dots. The SPN was the same whether symmetry and noise dots were segregated by color (e.g., symmetry red and noise green), or whether symmetry and noise were unsegregated by color (e.g., symmetry red and green, noise red and green). Both these segregated and unsegregated symmetry SPNs were stronger than the anti‐symmetry SPN. It seems that the extrastriate symmetry network is flexible enough to feed on a range of color or luminance‐defined features. However, it prefers such features to be matched at symmetrical positions (see also Gheorghiu et al., 2016 for equivalent behavioral results and interpretation).

Wright et al. (2018) also considered SPNs generated by polarity‐grouped anti‐symmetry, where all elements on one side are black, and all elements on the other side are white. This generated a very similar SPN to symmetry, and a larger SPN than conventional anti‐symmetry. Polarity‐grouped anti‐symmetry is thus more salient than other forms of anti‐symmetry. This requires further research.

Different theories of symmetry perception are couched at different levels of abstraction and vary in their generality and neural plausibility (Bertamini et al., 2018). One family of theories can be described as filter models (Dakin & Herbert, 1998; Dakin & Hess, 1997; Dakin & Watt, 1994; Osorio, 1996; Poirier & Wilson, 2010; Rainville & Kingdom, 2000; Scognamillo, Rhodes, Morrone, & Burr, 2003). Since pioneering work in the 1960s (Campbell & Robson, 1968) low‐level vision has been conceptualized as an array of retinotopically arranged filters with orientation and spatial frequency tuning. Rainville and Kingdom (2000) argued that good theories of symmetry perception should use this dominant paradigm as their starting point. One example is the *blob alignment* model introduced by Dakin and Watt (1994). According to this model, symmetry perception begins with low‐pass filtering of the image. If reflectional symmetry is present, filtering produces colinear aligned blobs which straddle the axis. Symmetry discrimination can then be based on blob alignment (see also Dakin & Herbert, 1998; Dakin & Hess, 1997). Other work has shown that symmetry discrimination is unaffected by overlaid random noise masks, but only when they differ from the underlying symmetrical pattern in terms of spatial frequency (Julesz & Chang, 1979) or orientation (Rainville & Kingdom, 2000). This suggests symmetry perception uses information derived from spatial frequency and orientation tuned channels.

Our current results show that filter models cannot ignore anti‐symmetry on the grounds that it is only detected through selective attention. Perhaps filter responses might be half‐wave rectified to give luminance polarity‐independent signals (Mancini et al., 2005)?

Another family of theories are cognitive/perceptual models, such as the holographic weight of evidence model (van der Helm & Leeuwenberg, 1996). This provides as a formal account of the fundamental ‘holographic identities’ which make up a visible 2D regularity. Subjective salience of the regularity (W) equals the number of holographic identities (E) divided by the total number of elements (N). The W = E/N formula successfully predicts SPN amplitude (Makin et al., 2016) and detection speed (Nucci & Wagemans, 2007) across a range of different visual regularities. However, the formula only explicitly considers position and numerosity information, not the additional role of luminance (mis)matching. In future, the holographic approach could be adapted to down‐weight different kinds of anti‐symmetry appropriately.

## 5 CONCLUSIONS

Here, we have shown that a standard form of anti‐symmetric dot patterns is extracted from the image automatically, even when it is not task relevant. This is an important consideration for future models of symmetry perception, which must be flexible enough to accommodate anti‐symmetry, while also accounting for its relative perceptual weakness.

## CONFLICT OF INTEREST

We declare no conflict of interest.

## AUTHOR CONTRIBUTIONS

Alexis Makin designed the experiment and facilitated data collection, then analyzed the results and wrote the manuscript. Marco Bertamini obtained funding for the project, and contributed to study design, interpretation of results and writing of the manuscript. Giulia Rampone assisted with EEG analysis techniques and interpretation of results.

## DATA AVAILABILITY STATEMENT

All ERP and behavioral data, and codes for analysis and stimulus presentation, are freely available on Open Science Framework: https://osf.io/yjg9q/. We are happy for other researchers to use this material.
